# University Gynaecology and Obstetrics, quo vadis? A Department of Women’s Health—University Women’s Hospital of the future?

**DOI:** 10.1007/s00404-014-3401-7

**Published:** 2014-08-19

**Authors:** Elisabeth Simoes, Sara Y. Brucker, Bernhard Krämer, Diethelm Wallwiener

**Affiliations:** 1 Centre of Women’s Health, University Hospital of Tuebingen, Calwer Str. 7, 72076 Tuebingen, Germany; 2 Women’s Health Research Institute, Calwer Str. 7, 72076 Tuebingen, Germany; 3 Staff Position Social Medicine of the University Hospital of Tuebingen, Calwer Str. 7, 72076 Tuebingen, Germany

**Keywords:** Women's Health, Department, Women's Health Research Institute, University Gynaecology and Obstetrics, Women's Academy

## Abstract

**Purpose:**

Numerous changes in society, science and health care challenge gynaecology and obstetrics. These challenges include the maintenance of excellence in research, commercial potential and clinical innovation, as well as the maintenance of adequate human resources, new standards for patient orientation and individualised medicine.

**Methods:**

Based on a SWOT analysis of the status quo, of local and national quality data, a search regarding national conceptions and of international best practice for women’s health centres, the model of a Department of Women’s Health was developed.

**Results:**

The Department, consisting of a University Hospital and a Research Institute, should interlink clinical care and science. With the establishment of the department, a pool of expertise is achieved which encompasses gynaecology and obstetrics from basic care to the high-technology segments, as well as all the scientific areas relevant to the medical discipline and women’s health, including health services research. Preservation and attraction of personnel resources are based on the department’s excellence, on reliable perspectives and the flexibility of job profiles, which also result from the close connection between care and research and the expansion of perspectives on women’s health.

**Conclusions:**

Methodological diversity and inter-professionalism build the appropriate base for the further development of research fields. At the same time, the Department creates space for the consolidation of the core areas and the integration of sub-disciplines (clinical and scientific) to maintain the unity of this discipline. Via the scientific monitoring of the implementation, suitable elements can be highlighted for transfer to other facilities.

## Introduction

Over the last few years, gynaecology and obstetrics—as other medical specialties—have been subject to extensive changes. Increasing specialisation of techniques, methods and procedures [1, 2] have been observed in diagnostic and therapeutic health care, as well as in research. This development will also continue over the coming years. With this, there is a growing risk that the professional unity will be fragmented and that isolated innovation processes will not be integrated and implemented in the speciality.

At the same time, clinics and research facilities are under increasing pressure to perform and under increasing competitive pressure to be able to exist under clinical, scientific and economic terms and to continue to develop. The condensation of demands on hospitals observed in all industrial nations also characterizes the situation in German women’s hospitals [3, 4]. Based on these aspects, a pooling of expertise and resources in the areas of science and health care appears sensible.

Furthermore, the focus is shifting towards awareness of health-related opportunities against a background of demographic change. Treatment of illnesses thereby represents a particular and still very important function, but not the sole function of medical practice. For obstetrics and gynaecology as a specialist area, this signifies a broadening of perspectives towards women’s health.

A variety of adaptations and the further development of structures in the specialist area are necessary to face the actual and the future challenges for health care and research arising from the rapid change through the ages [5].

## A model

### Questions and methods

In this context, the relevant questions are: how can the unity of the speciality survive and all facets of the specialist area be secured as part of a union? How can the knowledge that the speciality is committed to holistic gynaecology be implemented practically? How can the paradigm shift of bringing women’s health to the focus of attention be accommodated, and how can this mission be realised as a priority? University women’s health is committed to excellence and international prominence, and new processes and structures must support it.

One method of doing this is the expansion of obstetrics and gynaecology-focused institutions into Departments of Women’s Health. The basis for this should be the integrative merging of a University Women’s Hospital with its respective excellent standard in diagnosis, treatment and care with a Women’s Health Research Institute, which complements the department’s profile with its variety of approaches to research and health care, and develops the areas of knowledge management, prevention and health promotion.

The present paper initially presents the concept of a department for women’s health as a model for university institutions. The various components of the concept tie in with current challenges. The elements were prepared on the basis of a SWOT^1^ analysis. This included quality data (on processes, structure and results) from the University Women’s Hospital in Tuebingen, as well as benchmarking results presented in the literature, national quality reports, analyses and concepts for the further development of this specialist area and an investigation into international best-practice models for the design of departments and centres for women’s health. The results are presented in the following section with regard to the problem areas (weaknesses, threats) of the current situation. Additional benefits and importance for the specialist area are finally discussed (Sects. “Vision—Department of Women’s Health”, “Additional benefit for the medical speciality and University Medicine) in view of the synergy effects for the preservation and expansion of competence and excellence (strengths, opportunities), which should develop at all levels.

### Implementation and evaluation

The concept is presented as a model. Various components are in the process of being implemented at the Tuebingen University Hospital within the University Women’s Hospital and the associated Women’s Health Research Institute. The experience already gained from the previous development of these institutions will be reported. The implementation of the overall concept within the present Department of Women’s Heath of the University of Tuebingen is aimed for, supported ministerially and by the Faculty of Medicine. Using accompanying research, the exemplary structure of a department at a university institution can prepare evidence for the transferability to academic institutions and elements for the transfer to other obstetrics and gynaecology institutions. This will be achieved by the implementation of an international quality advisory board with the participation of the specialists, medical societies and professional organisations. Evaluation is planned in two topic areas: as process evaluation with reflective parts, which continues to shape the concept in parallel to implementation (monitoring and feedback). In the concluding phase of a chosen model period of time, whether and to what extent the expected outcomes occurred can finally be measured in the summative evaluation.

## Status quo and future challenges

### The current situation involves risks

Actual analysis of the situation of University Women’s Hospitals in Germany showed that on various occasions, the balance between the clinical strength of a department and research is not achieved in all areas. There is often a need for additions—structural and personnel. The interlinking of activities in medical care and research is not sufficiently developed. In some areas, an imbalance between clinical, translational research and fundamental research is also observed. In the case of inhomogeneity, university-internal and -external awareness of the research-clinical positioning can be unfavourable for the institution’s attractiveness.

Amongst the specific problem areas is the fact that a next generation which is qualified in the entire spectrum of the subject is largely absent. A Europe-and Germany-wide shortage of management personnel with clinical and research expertise has been observed [5]. This fact can be considered a threat for the continuity of highly qualified gynaecological health care in Germany, as well as for the progress of research. Consolidation or supplementation of the corresponding positions at the institutions is necessary, so that all clinical facets of the profession and areas of research are similarly covered, and their interlocking leads to outstanding results. However, the successful recruitment and the retention of qualified experts at an institution require suitable perspectives. These are indispensable when sustained staffing is sought. Many medical director posts and even professorships cannot be adequately filled at the moment. About one-third of posts in obstetrics and gynaecology are filled by part-time staff. From the view of society as a whole, a further increase in demand for part-time jobs is to be expected (in the light of duty of care in the family environment and demographic change [6]). Discontinuity in the development of expertise is to be feared, caused by short-term filling of positions, fixed-term employment and precarious employment positions. At the same time, there is a continuous threat of leading clinicians and researchers being headhunted. “Brain drain”, which has a particular impact on the medical profession, and inadequate support structures for highly motivated doctors (also with part-time positions) lead to further loss of stability and excellence, i.e. the performance capability and financial power for the alliance of a university hospital. Diversified but existent potentials call for a change regarding the job profiles and the educational structures the units have to offer.

For individual clinics, there is also an area of conflict between the tasks as regional medical centre and cutting-edge research. As a result of this conflict, there is a consecutive financial problem for the affected institution, as the allocation of performance-related resources is not adequately represented in relation to the total budget. The health care mandate of such clinics requires separate appraisal in the future.

Lack of inclusion of research topics, for example those which overlap other disciplines (social sciences, law, genetics etc.), drain clinical research or fragment it in such a way that research for women´s health occurring outside the profession of obstetrics and gynaecology is no longer comprehensible and may be more present in other facilities, as can already be observed for the area of prevention. This development hinders prompt translation, as well as the orientation of research topics towards the perspective and experience of the clinic.

The traditional high demands of obstetrics and gynaecology clashes with the increasing orientation of clinical medicine towards product definition, e.g. Diagnosis Related Groups (DRGs). This neglect of other areas of medicine from predefined performance units such as EBM and DRGs affects, for example, the psychosocial needs of patients, the need for training and further education for junior staff in the speciality or the significance of the doctor–patient contact for the results of medical treatment [7]. The dispute of this neglect has to take place at the system level, first of all. But it is a current and urgent task field, not only in social medicine, but also—in terms of the future sustainablity—of every other clinical discipline.

### Challenges of individualised medicine

The future is said to lie in (biomarker based) personalised medicine. Based upon scientific progress regarding the investigation of a disease’s cause and the technological achievements of life science research, medicine that is tailored to the individual requirements of the patient seems possible. However, individualisation equally requires new benchmarks of assessment, as well as patient-orientated potential in diagnostics, technology, clinical services and communication. To avoid a biologisation of the understanding of disease [8, 9], which excludes the personal dimension of illness and suffering, appropriate structures are needed which provide enough space for the cooperation of research and clinic as well as direct doctor–patient contact. The individualisation signifies a change in mindset. New therapeutic concepts are sought, which can be used in a small patient group only, but should be even more effective here and associated with fewer unwanted side effects. This approach is attracting much interest and support [10]. For progress which is in accordance with the various needs of health care reality, diagnostics, therapy, clinical experience and research, especially health services research, have to go hand in hand.

This also applies to obstetrics, where advances are emerging in completely new terrain with incalculable consequences. The analysis of the embryonic genome can potentially drastically change prenatal diagnostics [2]. The discipline must be prepared for the effects and the sequelae—even to the point of ethical debate. An increasing need for interdisciplinarity of personnel is becoming apparent.

### Health and health literacy in focus

A further paradigm shift has moved health to the centre of attention, not only for commercial or societal interests, but also as a duty of medicine. The move towards shaping health for the future means that the profession must put women’s health at the centre of the speciality. This also includes the demand of equal health opportunities.

Women’s health is at the centre of the area of conflict between the changing position of women in society, the demographic development in conjunction with the economic changes in society and health care systems and the rapid advancements in the field of biomedical and medical technology innovations. Women’s health transcends the medical speciality of obstetrics and gynaecology; it is interdisciplinary and incorporates the sociocultural environment and social relations, and it is also currently at the centre of scientific topics against the background of the aforementioned changes.

The subjects in the national competency-based list of learning targets (NKLM, [11]) for training in human medicine, currently under process by order of the Standing Conference of the Ministers of Education and Cultural Affairs, reflect the change and refer to health orientation and promotion at different levels in the competence of medical doctors. The consolidation of the scientific competence of young academics is also desired.

## Vision—Department of Women’s Health

Considering the presented challenges and with the aim of sustainable consolidation of clinical excellence and the development of trendsetting future scientific units, with a focus on basic research, translational, clinical and health services research—in alliance with and reflecting the respective university speciality concepts—the establishment of Departments for Women’s Health offer a necessary platform for the integration of all demands. In this alliance, all components should complement one another to form an integral concept, at the focus of which are women respective patients and their health. The two central units are a University Women’s Hospital and a Women’s Health Research Institute. The department should interlock clinical care and research in such a way that a profile-building institution is developed from the synergy of both parts, which internationally represents obstetrics and gynaecology and women’s health and moves the topical issues of the university and the university hospital forward (see below).

The main ideas of the restructure are to allow the interactions between healthcare, clinically orientated translational and healthcare-research and basic research to become increasingly effective, to promote clinical and scientific competency and to jointly represent women’s health in academic teaching and other areas of knowledge transfer more prominently. Visibility in a department characterized as centre of excellence will move the scientific and social perception of women’s health further forward.

### Future framework

**Fig. 1:**
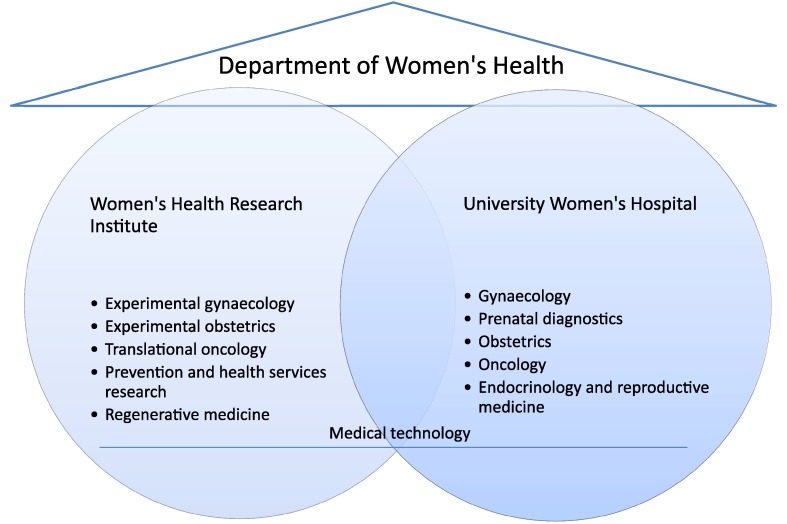
Functional diagram of the Department of Women’s Health: complementary interlocking of clinical unit and research unit

This extended requirement profile calls for new trendsetting structures for the University Women’s Hospital as a clinical unit, for the parallel complementary focused expansion of the Women’s Health Research Institute as the new department’s research unit and for the network. At the centre of the new basic structure is the grouping and complementary integration of health care at the University Women’s Hospital and research within the Women’s Health Research Institute under the roof of a Department of Women’s Health (Fig. 1). For this purpose, the further development of both central units towards a centre of excellence is required, as well as the establishment of a department which encompasses the organisational units “University Women’s Hospital” and “Women’s Health Research Institute“.

### Consolidation and optimisation of a University Women’s Hospital within the Department of Women’s Health

The first aim with regard to the university women’s hospital is to further strengthen its basic structure and significance as the department’s clinical unit—not least to preserve the performance of the university hospitals as a whole.

The risk that a university hospital loses highly qualified and clinically scientifically oriented management personnel should be strongly opposed by structural changes which increase the establishment’s attraction for long-term retention and to gain excellent obstetricians/gynaecologists and researchers. The basis for a top-ranking position in university medicine and science is the solid internal structure with a strong core clinic as a platform, with the central departments of gynaecology and obstetrics under singular management. The specialities of gynaecological oncology, obstetrics and perinatal medicine as well as endocrinology and reproductive medicine and further specialties build on this, endowed with corresponding professorships that represent scientific and medical substance (Fig. 3). This reflects the basic structure of the medical specialty, with room to supplement site-specific features.

The consolidation should be directly supported with the following actions:

- Research posts and professors for the core specialties of gynaecology and obstetrics at the University Women’s Hospital, to retain excellent clinically qualified experts who are qualified as professors at the institution or to newly enrol them.
- Endorsement of research posts for experts, who are attracted to the institution by the perspective of an international centre of excellence for women’s health, such as, for example, for prenatal diagnostics, specialised gynaecology, senology and others in which the special profile of the Women’s University Hospital is reflected.

This structural concept, which has also been implemented at the University Women’s Hospital in Tuebingen, corresponds with the DGGG recommendations for the development and structuring of university facilities. [12, 13]. Figure 3 shows an overview of this structuring and the University Women's Hospital as part of a Department of Women's Health.

### Development of a Research Institute for Women’s Health

**Fig. 2:**
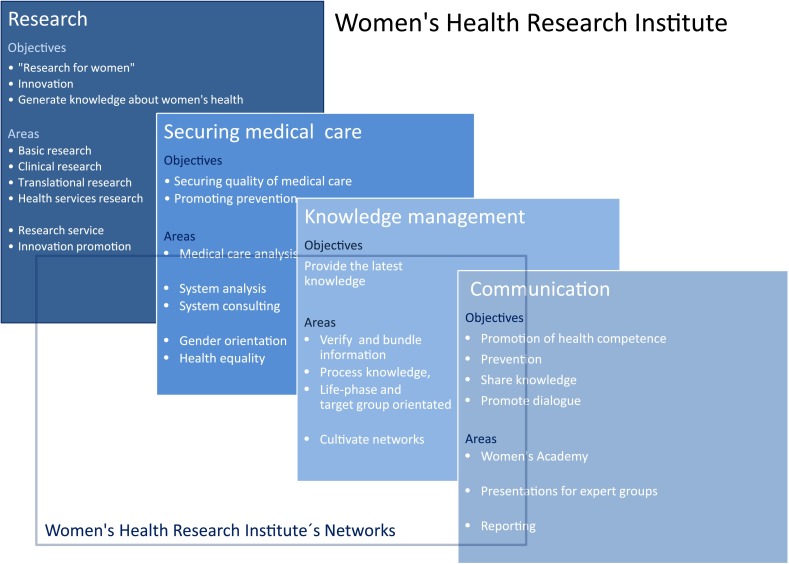
The activities of the Women's Health Research Institute at the University of Tuebingen act synergistically for the multidimensional promotion of women's health and offer the basis for translation and communication of expert knowledge for the layperson and expert groups. The methodical diversity and the interprofessional work groups of the research institute offer a suitable foundation for the further development of fields of research. It simultaneously creates space for setting individual priorities in sub-disciplines and for their integration into the department’s network

The aims when developing or expanding a research institute are interdisciplinary development, and the extension and deepening of basic research and translational, application- and healthcare-research in all areas of obstetrics and gynaecology. This can increase the desirability as a partner for other areas of research at the university, for external research facilities and for business companies, e.g., biotechnology. The research institute, positioned alongside an institution which has established itself excellently in patient healthcare, plays a key role for the increasingly important process of rapid transfer of research to use in clinical care. All areas of research for women are, therefore, at the focal point of the institute. Other main aims are the promotion of the concept of prevention, the strengthening of women’s health literacy, (both in society and in health care) as well as the securing of care, which incorporates quality assurance. A well-structured knowledge management serves all these areas. The activities complement one another in their contribution towards the promotion of women’s health. Figure 2 shows the various platforms of such an institute, using the Women’s Health Research Institute at the Tuebingen University Hospitals as an example.

Established with the aim of advancing research in women’s health and consolidating and circulating expert knowledge by the pooling of women’s health-related competencies, the Women’s Health Research Institute at the University of Tuebingen has experienced a successful evaluation from an international team of experts from the Baden-Wuerttemberg Foundation in cooperation with professional scientific associations (including the German Society of Gynaecology and Obstetrics and the German Federal Association of Obstetricians and Gynaecologists). The Institute has continued to evolve. The expertise available at the Institute goes beyond specialty-specific competencies and broadens horizons and fields of research, including qualifications and research in social medicine, occupational medicine, public health as well a multi-layered system- and methodological-competence. For example, one core area focuses on the task of the Women’s Health Research Institute that is directed towards prevention and health care. Health services research, which has increasingly greater significance for the shaping of the healthcare system—a fact also shown by the increasing consideration of this area in the (inter)national research funding—of particular importance. Health services research will form the centre for preventative medicine and gender-specific research for women in the future and accompanies areas of innovation, such as personalised medicine. The aim is to include clinical and interdisciplinary health services research in all research segments. This concept should lead to a rapid transformation of research results into progress in the field of health care and health care structures.

The implementation of new subject areas is an important contribution to research into women’s health. Actual and potential analyses of the respective institution show the need for development of areas with high potential. The selection results from the clinical units with their scientific excellence that already exists, the complementary development of the research unit as well as the perspectives of the new department. The following selection is not exhaustive, but provides an introduction:

- Experimental/translational gynaecology
- Experimental obstetrics
- Translational oncology with transregional joint professorships in close relation with the German Cancer Research Centre
- Prevention and health services research and women’s health
- Minimal-invasive therapy and technological processes
- Regenerative medicine with joint professorship with the Translational Fraunhofer Centre for Regenerative Medicine and Medical Technology

**Fig. 3:**
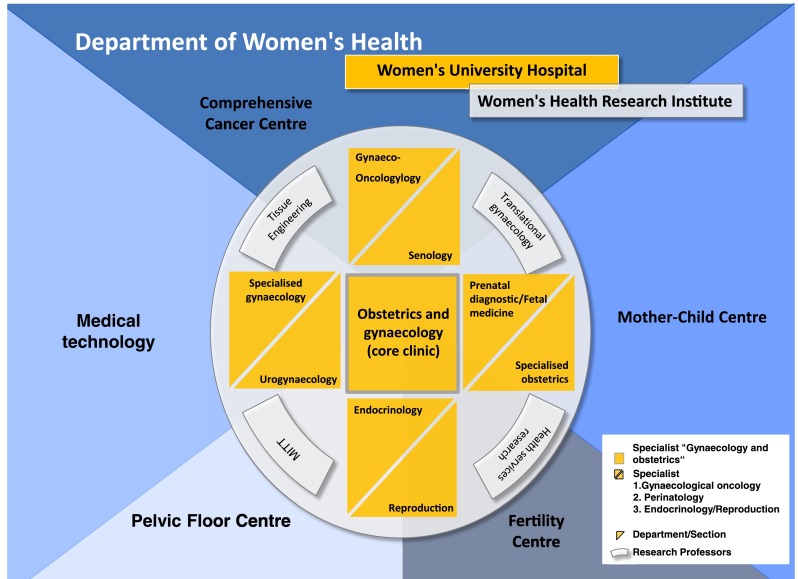
Internal structures of the Department of Women’s Health and external networking: The core clinic incorporates Obstetrics and Gynaecology, represented by professors. The areas of the speciality which require a special qualification complement the core clinic as well as the related sections. Research posts for experts and research professors in specialist areas substantially extend the department’s portfolio (for a more detailed explanation, see “Challenges of individualised medicine” and “Health and health literacy in focus”)

The synoptic integration is depicted in Figs. 1 and 3.

### Profile and significance of a Department of Women’s Health

A focus on clinical and scientific gynaecology and obstetrics has developed through the alliance of gynaecology and obstetrics with interdisciplinary research into women’s health research and the alliance of clinic and research. The establishment of a Department of Women’s Health further develops this leading position—in terms of an international centre of excellence for women’s health—with further complementary clinical and scientific units, which build on existing profile-building areas. In the future, research oriented clinical areas and healthcare-related research in the department should mutually support each other in all areas of further development, and thus advance the development of the centre of excellence.

An integrated health care chain with highs standards of diagnosis, treatment and care at one site is, therefore, complemented by healthcare- and patient-oriented basic- and applied-research at an international level. The substantial and strategic unity of research for women, which is specific for a University Women’s Hospital, will thereby be continued and further developed. With close interconnection of clinical and research units, the clinical topics are reflected in corresponding research modules (see below).

### New structural elements

A complementary formation with a focus on the new “organisational unit gynaecology and obstetrics” and “organisational unit Women’s Health Research Institute” is required for the development of a centre of excellence based on a departmental structure. The activities of the University Women’s Hospital and the Women’s Health Research Institute will be subdivided into modules in the future, producing a new complementary internal structure (Figs. 1, 3). The clinical modules reflect the structural arrangement of the gynaecology and obstetrics speciality. The research modules use this structure and supplement them. This modular structure provides the department with space for innovative processes, application areas and with new ways of using personnel resources (see below).

The structure and the contents of the department reflect the wide range that extends from basic research (e.g. genetics) to the research of the health care structure, embracing clinical studies for therapy optimisation as well as patient orientation (psychosocial/somatic aspects). Extensive knowledge is, therefore, built and the outstanding competence in the respective medical field is continually enhanced, represented by up-to-date health care and patient information. Results of research are rapidly transferred to the clinic and the need for further research will be brought into the research topics, based on clinical experience.

Certain investment funds are needed for the restructuring for:

1. a basic level of human resources,^2^ especially for the development of the Women’s Health Research Institute, including capital for facilities, consistent with the new requirements and structures,
2. a basic environment for various junior research groups, corresponding with the newly formulated clinical-scientific modules, which should be enabled to support specialist clinics amongst other things.

Junior doctor rotation between the Institute and the clinic anchors the research concepts in the clinic and vice versa.

At the same time, innovative job profiles arise meeting future demands. Part-time posts, research semesters (e.g. for challenging research proposals) and jobs with telestructure amongst others, may correspond to changing life phases and circumstances. The connection with the speciality remains and allows the medium- to long-term development of competence—with personal setting of priorities for each individual clinician and researcher, corresponding with the phases of his or her continuing education or career path. The discipline gains desirability and competence by the use of structures which allow completely new flexibility. The extent of this gain is set against the background of the recruitment situation of young professionals, but it is also a relevant competitive factor regarding the needs of a changing society. The department attains its possibility of flexibility from the close and complementary linking of clinic and science. Life-phase oriented change of priority setting in the clinic or in research, adaptation of working hours, which is rather more possible in the scientific context than in the clinic, and the creation of specialists, which can even result from part-time employment, represent just a small selection of forward-thinking options to strengthen the speciality.

The willingness to imagine and allow career development—contrary to the trend of previous years—even over longer periods, also calls for a revision of the research promotion and staffing policy of university institutions. Further education and competency development opportunities over longer periods are reflected in the new social demands such as “life-long learning“, or a longer working life, and require a shift away from paradigms of the irreversibility of work-family decisions of previous years. Occupational and public medicine research currently acknowledges part-time employment (often with family commitments such as child-bearing, long-term care of ill or disabled relatives, usually women) with a positive work relationship, particularly high motivation, identification with and loyalty to the employing institution [14]. Regardless of the many well documented benefits which institutions could draw from supporting policies, this path is still not sufficiently conceptualised [15]. The department creates innovative options by its modular structure.

An improvement of the study centre facilities and a staff position knowledge management will increase the responsiveness to emerging scientific developments. Alongside modules, which exclusively belong to the University Hospital for Women and those which exclusively belong to the Research Institute, there are also joint modules. Both institutions remain closely linked.

### Visibility and excellence in teaching and professional education

The academic teaching and traineeship of the discipline also has to face new challenges [16], one of which is the new competence based educational objective catalogue (NKLM) with focuses on competence fields, another is the enhancement of specialties within the field of obstetrics and gynaecology. A high rate of professional, didactic and social competence of the lecturers regarding the actual teaching assignment is required, as well as personnel resources for the specialties in clinic and research. Both elements are integrated with specific tasks which are defined in the department. This enhances the authenticity of teaching and traineeship, especially regarding the junior staff. This is achieved by supplementary aspects and issues that arise from a broader spectrum within the department, as well as by the involvement of renowned researchers in teaching who serve as a role model for students and young doctors interested in research.

The objective is to form a structured curriculum of modules which includes medical care in the hospital and the different topics of the research units, thus promoting the scientific qualification of the junior staff. This allows for an expansion of professional options which are adapted to different phases of life. The possibility of a Master’s qualification in new study courses (medical technology, molecular medicine and potentially other fields) can be established in the department. The emphasis of gender sensitivity in education is part of the fundamental issue towards a stronger individualization, which will be further described in this article.

Academic education based upon a department that *combines research and medical care* corresponds with the requirements of the national educational objective catalogue (NKLM) that seeks to strengthen the scientific competence of medical doctors by means of a comprehensive catalogue, extending from basic research knowledge to practical scientific studies. This corresponds to the intention to strengthen research from the centre of clinically practical medicine and requires adjacency to a research orientated unit. The Department of Women’s Health prepares this development. At the same time, the integration of student researcher extends research groups possibilities regarding for example an expansion of research topics or flexible time and project management.

The effect of the continuing comprehensive education concept is gathering impact. It shapes the practice of gynaecological care beyond the region, and is of importance for the preservation and development of referral structures. The proximity of clinic and research in the department is not only of interest and benefit to the admitting and referring partners (practices, ambulatory health care centres, clinics), but also to the patients. They can expect information about the latest scientific findings, involvement in trials, contact with researchers, etc.

## Additional benefit for the medical speciality and University Medicine

The department encompasses clinical care, research for women (from basic research to translation and health services research), prevention and health promotion, information and health education. It requires the substance of a Department of Women’s Health to be able to integrate all these perspectives in an overall concept and to thereby be able to face the challenges for health care and research evolving from the rapid changes in many fields.

### Unity of the speciality

Integration within the framework of a department which is focused on women’s health signifies an immediate strengthening of the discipline's unity at various levels:

- It reduces content-based and staff fragmentation as well as an impaired overview.
- It allows definition of research topics jointly by research and clinic, i.e. from clinical practice, where the awareness of areas requiring research develops directly.
- All (sub-) specialties are embedded within the union of the department by means of research posts and professorships.
- Definition of care management and quality by the department management comprises all facets of the speciality. This leads to a harmonised strategic orientation in the institution.
- Consistent interface definitions characterise the demarcation to affiliated subjects, research disciplines and areas of medical care.

The departmental structure creates possibilities for the innovative design of jobs and advanced training posts for better vocational bonding by means of perspectives and the security of an alliance. Academic institutions have a pacesetter role to also make this process efficacious in broader health care.

### Scientific collaboration partners and internationality

Women’s health is also an interdisciplinary challenge for science. This has to be accounted in the internal configuration of the department as well as the research cooperations. Implicitly, by means of different cooperations the department gains access to a broader spectrum of supporting institutions and subsidies, especially if these build on interdisciplinarity and multicentricity with respect to research issues. The Department of Women’s Health signifies an enhancement of the topic women’s health. This widens the horizon of existing networks, opens up new areas of research and increases the international significance of activity and results in the light of the increasing interest regarding gender sensitivity in research, education and health care.

**Fig. 4:**
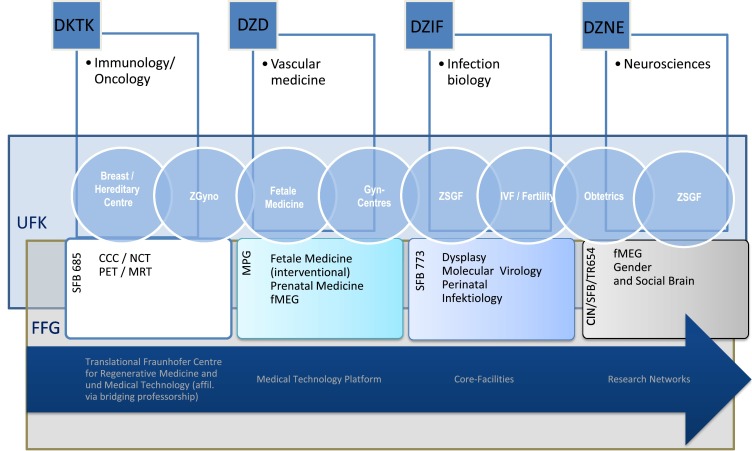
Research topics from the Tuebingen Faculty of Medicine and corresponding research priorities in the University Women’s Hospital centres and in the Women’s Health Research Institute: Immunology/oncology, vascular medicine, infection biology and neurosciences are at the centre of the research priorities in Tuebingen, reflected in four Helmholtz Association health centres. Health research at the Helmholtz Association, in which 18 natural science-technological and medical-biological research centres have merged, is addressing the causes of cancer, cardiovascular and metabolic disease, lung disease, neurological disease as well as infectious disease. This takes place in alliance with partners from University Medicine and Universities, of which the University of Tuebingen is also a representative for the depicted (sub-)specialties. Medical technology serves as linking scientific basis. Women’s health and obstetrics and gynaecology as medical disciplines are integrated accordingly into these topics. (Abbreviations: see index)

The completion of existing networks by the focus on gynaecology/women’s health within a new institution of excellence forms a vector for an enhanced international visibility of other research areas. The figure below illustrates this option using the University Women’s Hospital in Tuebingen and the Women’s Health Research Institute as an example, showing their networking with the scientific research priorities of the Tuebingen University Hospitals and the University of Tuebingen research associations. The promising field of (biomarker based) personalised medicine is highly dependent on a research environment anchored in a hospital and based on clinical experience. The research network of the University Women’s Hospital in Tuebingen with the Research Institute for Women’s Health (Fig. 4) serves as an example for this connection by presenting a bridging professorship for regenerative medicine (project in collaboration with the translational Fraunhofer Institute for Regenerative Medicine and Medical Technology).

### Gender sensitivity in research, teaching and care

The influence of gender differences in medicine is highly topical and is followed with particular interest in society and health policy. In 2002, the WHO emphasised the importance of the gender based on biology and cultural norm for health and well-being [17]. Gender medicine which investigates and incorporates the sometimes great gender-specific differences regarding an illness’s symptoms, treatment and course is the first step to classify patient groups in the process towards personalised medicine. It is, therefore, currently a great chance to focus on women’s health. The department and the university will benefit from the high social acceptance that meets gender sensitive research, education and care as well as women’s health. As it has been on the edge area of research so far, very high potential for innovation, networking and expansion exists. This includes access to new subsidies.

Individualised medicine allows for a high treatment quality for the patients and opens up ways of cost-effective use of resources. For example, gender-orientated management of pharmaceuticals increases efficiency and thereby avoids false supply and allocation. In addition, this broad approach within a department allows going beyond personalised medicine that is only based on biomarkers; it can, therefore, offer the patients “individualised” medicine in the right sense of the word. For this purpose, the inclusion of other competencies such as social and psychosomatic medicine in the interdisciplinary research institute is necessary, to provide space for patients' self-determination and gender-equitable communication [18, 19].

## Discussion

Even if there are different paradigm shifts in medicine, the entire skills which exist in the speciality of gynaecology and obstetrics provide a basis that maintains the leading role for the specialty according to its competence: If the speciality wants to rise to the challenge of shaping the future of (women’s) health, this denotes a comprehensive move towards women’s health. The development of Departments of Women’s Health represents a structured setting of the future strategy in this process. The breadth of the speciality poses great challenges not only for gynaecologists undergoing training and further education, but also for extensive medical care [5]. Some aspects of this strategy are highlighted and discussed below.

### Securing locations and perspectives

Opportunities arise through integration, directionality and synergies. The alliance of a University Women’s Hospital with a Research Institute creates the most important basis for the future complementary development of clinical care and research in all the relevant areas of gynaecology and obstetrics and women’s health. This demands the strengthening of existing and the development of vacant scientific fields to columns of a modular alliance. Excellent clinicians, who are also designated as being excellent in their field of scientific expertise, will be interested in this concept. It could, therefore, open middle-term perspectives, as the extended possibilities of a department also offer working positions with a long-term orientated profile. This allows flexibility, orientated towards today’s increasingly varied demands. If necessary, rotation between areas of work within the department can be arranged with minimal effort and adaptation to the doctor’s own personal situation and range of interests (different ages) are possible, without forsaking the link with the establishment. This is due to the fact that personal time limitations interfering with employment, a scientific career or an engagement in the hospital are usually only temporary. Distinguished experts for specialist areas can be acquired and also retained. Provided that they are designated personalities of cutting-edge research, a catalysing effect on the further development of gynaecological care can be expected. Research results are the guarantee for the sustainability of the qualitatively superior clinical performance of university medicine in the field and for innovative health care for women. University Women’s Hospital and the Research Institute together can continuously built up research networks. They are the reliable basis for interdisciplinary and multicentric research tasks.

The central incentive still is the existing clinical and scientific reputation. However, this has to be complemented by solid offers which are precise and future shaping for the individual researcher. If a high clinical and scientific reputation exists, an international presence of the different specialities, as well as a high patient volume, then the attraction for young academics is increased.

A research structure that provides specialty areas and professorships that serve as bridges to other research fields (e.g. Helmholtz centres or interdisciplinary subjects within the faculty) forms complementary and substantial columns. Further interactions for special interests and competences are offered by the new areas of medical technology and tissue engineering (see below), as well as other research consortiums that are already established at the respective university hospital.

Another aspect for the recruitment of junior staff members is associated with this: Alongside the prospects of gaining exceptionally qualified clinicians and researchers attracted by the form and the concept of a department as described earlier, aspects of planning reliability and sustainability of the profession are to be considered. When asked about relevant factors for career choice and subject choice, both aspects were ranked first in a survey of medical students at the Heidelberg University Hospitals [20]. The presented advantages that follow from the broadness and stability of a department structure thereby strengthen the future prospects for the speciality when young academics choose their options. This study emphasises the importance of the doctor for good patient care, as does a current study by the University of Goettingen [21]. This indicates that patients' ability for self-determination develops during the doctor–patient contact and, from the patient’s perspective, the doctor plays a key role. Health-related knowledge and general coping skills can be best perceived by the patient in the actual situation of a doctor–patient relationship and specific health issues can then be translated into self-determined decisions.

### The extended (self-) demand of the subject: focus health

Curative medicine alone is becoming increasingly inadequate to counteract the consequences of chronic illness. This also applies to gynaecology. Prevention- and rehabilitation services are necessary to guard against illness, to reduce consequences of illness, and especially to ensure the participation of the affected persons in a job and in society. According to an evaluation by the Advisory Council in Health Care in their special report “Coordination and integration—health care provision in an ageing society”, age-specific prevention and health promotion is of particular importance [22]. If the individual risk of disease can be more accurately predicted and undesirable side effects and inefficient treatment strategies can be avoided, as expected from advances in personalised medicine, this would expand potential in the field of early recognition of illness and disease prevention.

In the 1970 s, the traditional system of health care provision was already criticised as being too organ- and symptom-related and as being based on a mechanistic understanding of disease. A consequence of the criticism of the mechanistic understanding of disease was the development of a bio-psychosocial model of disease. Somatic as well as psychological and social factors were referred to explain and treat illnesses. At the same time, efforts to prevent illness were intensified and amongst other things, this led to the concept of health promotion [23, 24]. The development of greater understanding of disease and health led to a differentiation of the scientific specialties which dealt with health issues, such as social medicine, medical psychology, psychosomatic medicine, psycho-neuroimmunology or health psychology. Depending on the profile, these disciplines can be integrated into the overall concept and orientation of the department by a Women’s Health Research Institute (including their research portfolio), and either be established there or be included in research cooperations, as shown by the example of the Women’s Health Research Institute at the Tuebingen University Hospitals. (cf. Figs. 2, 4).

### Women’s health goes beyond gynaecology

A department which is orientated towards women’s health revolves around the holism of gynaecology. It follows the Ottawa Charta [23], according to which health care promotion and self-determination regarding health issues are basic values for medical practice. Comprehensibility and coherence are of great importance for patients in an illness and recovery process, as has been increasingly demonstrated in scientific studies—not least for the quality of the coping process and life with or after the illness [25, 26]. Incorporating these aspects from the outset into the treatment offers and support for patients, wider structures are required as they can be provided in departments. (Psycho-)social, onco-psychological and rehabilitative specialist consultations or cooperations can be borne in mind, as can information offered by the Research Institute’s knowledge management, and a Women’s Academy [27] for the advancement of health literacy, for example. Alongside other issues, e.g. economic components of health, the United Nations singled out health equality as being of great social value in their agenda, “Advancing the Global Health” [28]. The promotion of women’s health fulfils this goal. The NKLM list of learning targets (see above) for academic education also emphasises the development of knowledge of different health components such as risk factors, the importance of personal resources for the overall health condition, the model of salutogenesis. Similarly, prevention and health promotion are increasingly found amongst the tasks that fall under the competence of medical doctors [29].

## Conclusions and outlook for practice

The presented strategy allows for a step by step implementation of the relevant processes. The speed of implementation as well as selected tasks that should preferably be modified can be orientated towards the requirements of the respective locations. Addition of further competencies, e.g., for the interdisciplinary staffing of the Research Institute, can take place gradually and be geared towards the development of the corresponding clinical or research areas. In Tuebingen, an interventional multiphase concept has been chosen, and the development of the Research Institute parallel to the advancement of the internal structure of the University Hospital for Women is currently underway. The consolidation of the specialities within the University Hospital for Women is a main priority. This process will be described separately.

The modular structure of a department with clinical and scientifically orientated components will also allow improvements in the interconnection of inpatient and outpatient sectors, also personnel. This can attain a trendsetting character, for example in the design of an area of expertise (e.g. specialist outpatient care according to § 116b SGB V [30], in conjunction with the statutory health insurance physician amending act [31]).

The paradigm shift in many areas of medicine towards increased attention to health, the move towards health promotion and the increasing expectations against the background of changing conditions in out patient and clinical care lead to the demand for facilities which are equally dedicated to women’s health in care, research and the advancement of health literacy for women. In addition to this is the social demand for flexibility. The basis for this should be the integrative alliance of a University Women’s Hospital with an outstanding standard of diagnosis, treatment and care and a Women’s Health Research Institute, which uses a variety of approaches in research and health care to complementarily broaden the spectrum of the department and the fields of prevention and health promotion.

The university institutions can perform the first exemplary step here. Owing to their particular importance for research, the creation of Departments of Women’s Health can initially lead to a close interaction between clinic and science. Further areas, such as student teaching, health information and continuing education will also profit from the extended competence, just as (interdisciplinary) cooperation within the inpatient and with the outpatient sector.

With the creation of Departments of Women’s Health, sustainable, strengthened institutions should develop, which can cope with societal demands and challenges. In the unity of a department all components complement each other to a uniform concept that exceeds the structures of the single units. At the centre of this concept is the woman respective patient and, therefore,—in the literal sense—individualised medicine.

## Abbreviations

CCC/NCT: Comprehensive Cancer Centre Tuebingen/National Cancer Centre, Heidelberg
CMV: Cytomegalovirus
DfF: Department of Women's Health
DGGG: German Society for Gynaecology and Obstetrics
DKTK: German Consortium for Translational Cancer Research
DZD: German Centre for Diabetes Research
DZIF: German Centre for Infection Research
DZNE: German Centre for Neurodegenerative Diseases
fMEG: Fetal magnetoencephalography
IZST: Interuniversity Centre for Medical Technology Stuttgart –Tuebingen
KFO 273: Treatment of urinary incontinence using cell based regeneration of the urethral sphincter
MPG: Max-Planck Institute
NMI: Natural and Medical Sciences Institute at the University of Tuebingen
SFB 685: Special research field: immunotherapy: from molecular basis to clinical use
SFB 773: Special research field: treatment resistant of solid tumours and how to overcome it
SFB/TR 654: “Plasticity and sleep”
Transregio SFB 654: “Plasticity and Sleep”
UKT: Tuebingen University Hospitals
ZSGF: Centre for rare genital malformations in women

## Footnotes

1 SWOT: Strengths, weaknesses, opportunities, threats.

2 E.g. Staffing expenditure for medical director, secretarial support and central staff, of which some are full time researchers, interdisciplinary arrangement.
